# Nanostructured Solar Cells

**DOI:** 10.3390/nano6080145

**Published:** 2016-08-09

**Authors:** Guanying Chen, Zhijun Ning, Hans Ågren

**Affiliations:** 1 School of Chemistry and Chemical Engineering, Harbin Institute of Technology, Harbin 150001, China; 2 Institute for Lasers, Photonics and Biophotonics, University at Buffalo, State University of New York, Buffalo, NY 14260, USA; 3 School of Physical Science and Technology, ShanghaiTech University, Shanghai 200031, China; 4 Division of Theoretical Chemistry and Biology, School of Biology, Royal Institute of Technology, SE-10609 Stocholm, Sweden

**Keywords:** photovolatics, solar cells, nanostructures, nanomaterials

## Abstract

We are glad to announce the Special Issue “Nanostructured Solar Cells”, published in Nanomaterials. This issue consists of eight articles, two communications, and one review paper, covering major important aspects of nanostructured solar cells of varying types. From fundamental physicochemical investigations to technological advances, and from single junction solar cells (silicon solar cell, dye sensitized solar cell, quantum dots sensitized solar cell, and small molecule organic solar cell) to tandem multi-junction solar cells, all aspects are included and discussed in this issue to advance the use of nanotechnology to improve the performance of solar cells with reduced fabrication costs.

With the growth of the world economy, the ever-increasing requirement for energy resources has become one of the biggest challenges for human beings. Solar energy—due to its tremendous reserves and environmentally friendly character—is generally regarded as one of the most important renewable energy resources.

Huge progress has been made in the commercialization of solar cells in the past decade, under the support of government subsidy. However, the large-scale replacement of fossil fuels by solar cells requires a further decrease in cost. High energy output efficiency and low cost are two criteria in order to achieve this purpose. Nanostructured solar cells provide a convenient strategy to address both issues simultaneously. Firstly, nanostructure can be explored to enhance light harvesting capability and increase efficiency. Secondly, nanostructure allows the use of less material in device fabrication, which can further decrease the cost.

To date, significant efforts have been made to explore nanostructure’s ability to improve the light harvesting capability of solar cells. Especially for silicon solar cells, the use of nanostructure significantly decreased the consumption of silicon materials. It effectively reduced the cost of silicon solar cells, and commercialization has already begun. In this issue, Jia and coworkers integrated aluminum nanoparticles on silicon solar cells, which facilitate light trapping and bring increased device performance [1]. Shapter’s group studied the effect of the size of carbon nanotubes on the performance of carbon nanotube/silicon heterojunction solar cells—a new concept in silicon solar cells [2]. In addition to single junction solar cells, nanostructures can also be utilized for tandem solar cells to enable current match between separated cells. Bai et al. utilized silver nanoparticles for GaInP/GaInAs/Ge triple junction solar cells, resulting in improved photocurrent and device performance. The overall power conversion efficiency was over 30% [3].

Looking beyond traditional solar cells, the application of nanostructures can be used for emerging solar cell technologies. PbS quantum dots thin film solar cells have received great interest due to their solution processability and light harvesting capability in the infrared region. However, the light absorption of quantum dots’ exciton peak position is usually much weaker than that in visible region. Wang et al. designed a new nanostructure that can significantly improve the light trapping capability of the film [4].

Dye or quantum dots sensitized solar cells are deemed an important kind of low-cost solar cell. They usually apply a scattering layer to improve light harvesting capability. Jun et al. explored silver nanoparticles to improve light scattering and bring enhanced device performance [5]. Agren’s group utilized PbCdSe alloy quantum dots for quantum dots sensitized solar cells, leading to much improved photocurrent compared to the control device with Cd only [6]. Bouclé explored nitrogen-doped TiO_2_ for solid-state dye sensitized solar cells, which has the potential to further improve the light harvesting capability of the electrode [7].

For small molecular heterojunction solar cells, Yun et al. explored morphology control to optimize the nanostructures inside. Without the use of any additives, the efficiency of solar cells can go to 6.8% [8]. Li’s group developed fluorine-substituted dyes for small molecular heterojunction solar cells, bringing increased device performance compared to the control device [9].

Bulk heterojunction solar cell-based nanostructured quantum dots and polymer are regarded as another important alternative for organic polymer bulk heterojunction solar cells. Krueger et al. used a surface engineering strategy to modify the surface of quantum dots, bringing improved device performance [10].

Finally, Chen et al. summarizes the exploration of upconversion materials for efficiency enhancement in solar cells [11].

This issue integrates the most recent study of nanostructured solar cells, which allows readers to quickly follow the recent developments in this area.
